# Chrysin inhibited tumor glycolysis and induced apoptosis in hepatocellular carcinoma by targeting hexokinase-2

**DOI:** 10.1186/s13046-017-0514-4

**Published:** 2017-03-20

**Authors:** Dong Xu, Junzhe Jin, Hao Yu, Zheming Zhao, Dongyan Ma, Chundong Zhang, Honglei Jiang

**Affiliations:** Department of General Surgery, the fourth affiliated hospital of China Medical University, The No.4 Chongshan east road Huanggu District, Shenyang, 110032 LiaoNing China

**Keywords:** Hepatocellular carcinoma, Chrysin, Hexokinase-2(HK-2), Apoptosis, Tumor glycolysis

## Abstract

**Background:**

Hexokinase-2(HK-2) plays dual roles in glucose metabolism and mediation of cell apoptosis, making it an attractive target for cancer therapy. Chrysin is a natural flavone found in plant extracts which are widely used as herb medicine in China. In the present study, we investigated the antitumor activity of chrysin against hepatocellular carcinoma (HCC) and the role of HK-2 played for chrysin to exert its function.

**Methods:**

The expression of HK-2 in HCC cell line and tumor tissue was examined by western blotting and immunohistochemistry staining. The activities of chrysin against HCC cell proliferation and tumor glycolysis were investigated. Chrysin-induced apoptosis was analyzed by flow cytometry. The effect of chrysin on HK-2 expression and the underlying mechanisms by which induced HCC cell apoptosis were studied. In HK-2 exogenous overexpression cell, the changes of chrysin-induced cell apoptosis and glycolysis suppression were investigated. HCC cell xenograft model was used to confirm the antitumor activity of chrysin in vivo and the effect on HK-2 was tested in chrysin-treated tumor tissue.

**Results:**

In contrast with normal cell lines and tissue, HK-2 expression was substantially elevated in the majority of tested HCC cell lines and tumor tissue. Owing to the decrease of HK-2 expression, glucose uptake and lactate production in HCC cells were substantially inhibited after exposure to chrysin. After chrysin treatment, HK-2 which combined with VDAC-1 on mitochondria was significantly declined, resulting in the transfer of Bax from cytoplasm to mitochondria and induction of cell apoptosis. Chrysin-mediated cell apoptosis and glycolysis suppression were dramatically impaired in HK-2 exogenous overexpression cells. Tumor growth in HCC xenograft models was significantly restrained after chrysin treatment and significant decrease of HK-2 expression was observed in chrysin-treated tumor tissue.

**Conclusion:**

Through suppressing glycolysis and inducing apoptosis in HCC, chrysin, or its derivative has a promising potential to be a novel therapeutic for HCC management, especially for those patients with high HK-2 expression.

## Background

Numerous mutations in oncogenes and tumor suppressors, as well as the alterations in gene expression profiles, result in the deregulations of cell metabolism in cancer cells. The Warburg effect, also named aerobic glycolysis, which is observed by Otto Heinrich Warburg in 1920s, is one of most prominent hallmarks of cancer cells [1]. In Warburg effect, even in the presence of sufficient oxygen, glucose is converted to lactate, instead of totally oxidized via Krebs cycle to generate ATP [2]. Tumor glycolysis supplies abundant energy to sustain the rapid tumor growth, moreover, the product of glycolysis such as lactate also provides an appropriate microenvironment to promote tumor metastasis [3]. So far, various regulatory pathways involved in the conversion from the Krebs cycle to tumor glycolysis have been well investigated, HKs were considered to be one of the most important effectors [4, 5].

As important glycolytic enzymes, HKs are responsible for the first rate-limiting step in the process of glucose metabolism, in which glucose is phosphrylated to glucose-6-phosphate. Four different isoforms of HKs, named as HK1-4 have been identified so far. HK-1 and HK-2 are mainly located on the outer membrane of mitochondria, HK-3 is positioned in a perinuclear compartment, and HK-4 is in the cytoplasm. Localization to the outer membrane of mitochondria confers HK-2 the advantage to escape product inhibition and gain preferential access to ATP in mitochondrion [6]. Among these different HKs, HK-2 is found to be expressed of high rate in malignant tumors and plays a key role in the development of Warburg phenotype. Overexpression of HK-2 was observed in various cancers, such as gastric [7], ovarian [8], breast cancer [9], cervical carcinoma [10], esophageal adenocarcinoma [11] and nasopharyngeal carcinoma [12].

Chrysin is a bioactive flavone derived from plant extracts such as blue passion flower, propolis, and honey, which are widely used as herb medicine in China. Except its multiple bioactivities in antioxidant [13], anti-inflammatory [14] and antibacterial [15], its antitumor potential is also well validated in a variety of human cancer cell lines. Chrysin is demonstrated to exert antitumor effect by inducing cell cycle arrest and apoptosis through different mechanisms, for instance, activation of extrinsic apoptosis pathway [16], alteration of cyclins and CDKs [17]. Moreover, multiple signaling pathways in cancer cells, such as Ras-Raf-MAPKs, PI3K-Akt, STAT, NF-κB, Wnt-β-catenin and Notch signaling pathways, were proved to be modulated by chrysin to inhibit cell proliferation, angiogenesis, invasion and metastasis [18–20]. However, the effect of chrysin on tumor glycolysis is largely unknown. Herein, we showed that chrysin inhibited glycolysis and induced apoptosis in HCC cells in vitro and in vivo. Mechanism investigations revealed that the biological activities exerted by chrysin were mainly attributed to its effect on HK-2. With the decrease of HK-2, chrysin inhibited tumor glycolysis and activated mitochondria-associated apoptosis. Given HK-2 was found to be overexpressed in the majority of HCC tissue, the results of present study suggested chrysin, or its analogues, may serve as effective candidates for HCC management.

## Methods

### Cell line and reagents

Chrysin and other chemical reagents such as Tris, NaCl, SDS and DMSO were purchased from Sigma-Aldrich (St. Louis, MO). The normal human hepatic cell LO2 and HepG2, Hep3B, Huh-7, HCC-LM3, Bel-7402 and SMMC-7721 were obtained from the Cell Bank of Chinese Academy of Sciences and cultured with Dulbecco’s Modified Eagle Medium containing 10% FBS and 1% antibiotics in a 37 °C incubator with 5% CO_2_. Anti-HK2, anti-VDAC1, anti-cleaved-caspase3, anti-cleaved-PARP, anti-cytochrome C, anti-Bax, anti-Bak, anti-Bcl-2, anti-Bcl-xL, anti-rabbit IgG-HRP and anti-mouse IgG-HRP antibodies were products of Cell Signaling Technology, Inc. (Danvers, MA). Anti-α-Tubulin antibody was purchased from Santa Cruz Biotechnology (Santa Cruz, CA, USA). Anti-β-actin (A5316) was product of Sigma (St. Louis, MO, USA). HK2 (ORF004940) construct was purchased from Applied Biological Materials (ABM) Inc. (Richmond, BC, Canada). Lipofectamin 2000 was purchased from Invitrogen (Carlsbad, CA).

### Cell proliferation assay

Human HCC cells (3× 10^3^ per well) were seed in 96-well plate and then treated with or without different concentrations of chrysin for 0, 24, 48 and 72 h. Cell viability was measured with Cell Titer-Glo Luminescent Cell Viability Assay kit from Promega Corp. (Madison, WI) according to the manufacturer’s protocol.

### Western blotting

Cells were harvested by trypsinization and pelleted by centrifugation. The pellets were lysed with RIPA buffer supplemented with protease cocktail (Roche, Germany). Protein concentrations in cell lysates were determined with the Bradford assay (Bio-Rad, Philadelphia, PA, USA). The lysates were subjected to SDS-PAGE and then electrically transferred to PVDF membrane (Millipore, Billerica, MA, USA). After blocked with 5% non-fat dry milk, the membranes were incubated with specific primary antibodies overnight at 4 °C. After washed with TBS-Tween 20 three times, HRP-conjugated secondary antibodies were incubated at room temperature for 1 h. The membranes were washed with TBS-Tween 20 and the bands were visualized using ECL chemiluminescence reagents (Pierce Chemical Co., Rockford, lllinois, USA).

### Apoptosis assays

Cells were seeded in six-well plate and then treated with chrysin for 24 h. After treatment, attached and floating cells were harvested by centrifugation. For apoptosis analysis, the cells were re-suspended with PBS and adjusted to 1 × 10^6^ cells/ml, then 5 μl Annexin V and Propidium Iodide staining solution were added to 300 μl of the cell suspension. After incubated 10–15 min at room temperature in the dark, stained cells were assayed and quantified using a FACSort Flow Cytometer (BD, San Jose, CA, USA).

### Glucose uptake and lactate production measurement

Glucose uptake and lactate production measurement were performed as previously described [21]. Briefly, Cells were trypsinized and seeded in 6-well plates (5 × 10^5^), after incubation for 10 h, cell culture medium was discarded and replaced with fresh culture medium containing different concentrations of chrysin for 8 h. Glucose and lactate levels were measured by using the Automatic Biochemical Analyzer (7170A, HITACHI, Tokyo, Japan). The relative glucose consumption rate and lactate production rate were normalized by the protein concentration of samples.

### Isolation of mitochondrial fractions

The cells (5 × 10^6^) from a 10 cm dish were harvested by trypsinization and centrifuged at 800 rpm for 5 min at 4 °C. The cell pellets were washed once with cold PBS and then resuspended with isolation buffer (20 mM Hepes, pH 7.4, 10 mM KCl, 1.5 mM MgCl2, 1 mM sodium EDTA, 1 mM dithiothreitol, 10 mM phenylmethylsulfonyl fluoride, 10 mM leupeptin and 10 mM aprotinin). After chilling on ice for 3 min, the cells were disrupted by 60 strokes of a glass homogenizer. The homogenate was centrifuged at 2,000 rpm for 10 min at 4 °C to remove unbroken cells and nuclei. The mitochondria-enriched fraction (supernatant) was then pelleted by centrifugation at 13,000 rpm for 30 min. The pellets was lysed in RIPA buffer (10 mM Tris-Cl (pH 8.0), 1 mM EDTA, 0.5 mM EGTA, 1% Triton X-100, 0.1% sodium deoxycholate, 0.1% SDS. 140 mM NaCl) and analyzed by western blot. For Immunoprecipitation, 500 μg extractions were pre-cleared with 30 μl (50% slurry) agarose A/G beads for 2 h rocking at 4 °C. The beads were removed, 30 μl (50% slurry) fresh agarose A/G beads and appropriate antibodies (4 μg) were added to the precleared lysate overnight at 4 °C. The beads were washed, mixed with 4 × SDS sample buffer, boiled, and then subjected to Western blotting.

### In vivo tumor growth assay

Six-week-old female Nu/nu athymic nude mice were maintained under specific pathogen free (SPF) condition in accordance with Institutional Animal Care and Use Committee. HCC-LM3 cells (5 × 10^6^ cell/mice) were subcutaneously injected into the right flank of nude mice. When the tumor volume reached about 100 mm^3^, the mice were randomly grouped, five mice per group. The vehicle group was given 0.5% sodium carboxymethylcellulose, and the other group received 30 mg/kg chrysin. Chrysin was administrated three times weekly via intraperitoneal injection. The tumors were measured twice per week using microcalipers and tumor volume(V) was calculated as following: V = (length × width^2^)/2. After the experiment, mice were sacrificed and the tumors are weighed and photographed.

### Immunohistochemical(IHC) staining

A human HCC tissue array (HlivH150CS03), which contains 75 cases of malignant HCC biopsy and 75 cases of matched adjacent normal tissue, was purchased from Shanghai Outdo Biotech Co., Itd. (Shanghai, China). Tumor tissues from xenograft model were fixed in 4% paraformaldehyde and then embedded in paraffin. IHC staining was performed as previously described [22]. Briefly, Paraffin sections were deparaffinized and hydrated, the endogenous peroxidase activity was blocked with 3% H_2_O_2_. Antigen retrieval was performed in citric acid solution using a microwave oven. The tissues were blocked with serum from the host of the secondary antibody and incubated with primary antibody of anti-HK2 (1:200) or anti-Ki67 (1:250) respectively at 4 °C overnight. Biotinylated secondary antibodies were added at a 1:100 dilution and followed by Vectastin ABC solution. Finally, the binding of the antibodies was visualized with 3, 3-diaminobenzidine (DAB) solution. Tissues were counterstained with harris’ hematoxylin and then dehydrated. Slides were viewed and photographed under a light microscope and analyzed using Image-Pro Plus software (version 6.2) program (Media Cybernetics).

### Statistical analysis

All statistical analysis was performed by SPSS software (version 13.0). All the quantitative data were expressed as mean values ± standard deviation, the significant difference between two groups was assessed by a two-tailed Student’s *t* test. *p* < 0.05 represented a statistically significant difference.

## Results

### HK-2 was highly expressed in HCC cell lines and tumor tissue

**Fig. 1:**
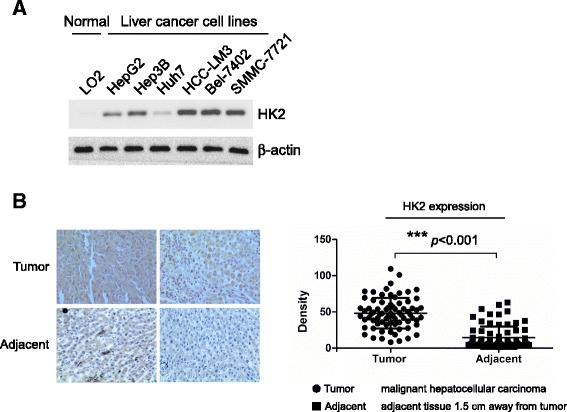
Aberrant expression of HK-2 in hepatocellular carcinoma(HCC). **a**, HK-2 was highly expressed in HCC cell lines. Western blotting was performed to examine HK-2 expression in several HCC cell lines and normal hepatic cell LO2. **b**, HK-2 was highly expressed in HCC tissue. Representative figures of immunohistochemical staining for HK-2 in HCC tissues and paired adjacent normal tissue (*left panel*), statistical results of HK-2 staining in 75 different HCC tissues and matched adjacent normal tissue (*right panel*). The asterisk (***, *p < 0.001*) indicated a significant difference of HK-2 expression between tumor and paired adjacent normal tissue

First, the expression of HK-2 was assessed by western blotting in six HCC cell lines. As the results shown in Fig. 1a, comparing to the normal hepatic cell LO2 in which no HK-2 expression was detected, at least five HCC cell lines (HepG2, Hep3B, HCC-LM3, SMMC-7721, BEL-7402) expressed high levels of HK-2. We next sought to examine HK-2 expression in HCC tissue and paired adjacent normal tissue. Consistent with the result in HCC cell lines, in matched normal adjacent samples, HK-2 was not detectable or at a relatively low level. In contrast, HK-2 was substantially overexpressed in tumor samples (*n* = 75, *p* < 0.001) (Fig. 1b). These results suggested that HK-2 might have a role in HCC development.

### Chrysin inhibited HCC proliferation and glucose metabolism

**Fig. 2:**
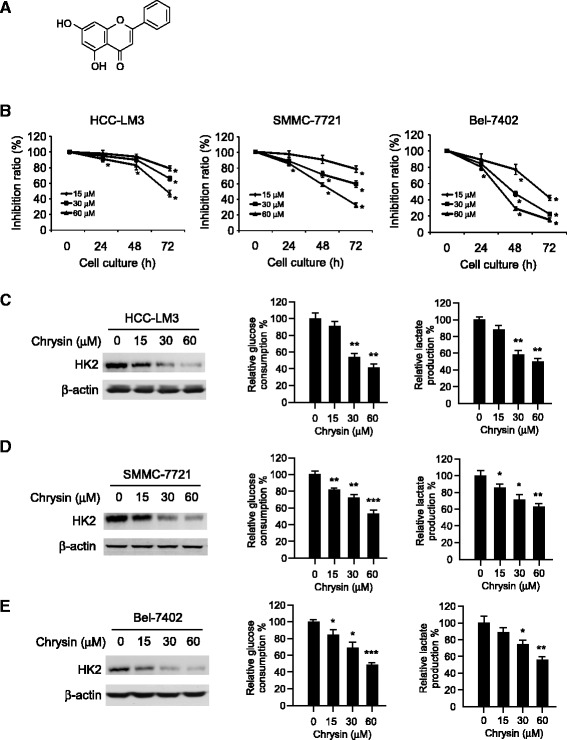
Chrysin inhibited cell proliferation and glycolysis in HCC cells. **a**, The chemical structure of chrysin. **b**, HCC cells were treated with indicated concentration of chrysin for indicated times, cell proliferation was measured as described in Material and Methods. The asterisk (*, *p < 0.05*) indicated a significant decrease of HCC cell proliferation after chrysin treatment. **c**-**e**, chrysin suppressed glycolysis in HCC-LM3 (*top*), SMMC-7721 (*middle*) and Bel-7402 (*bottom*) cells. HCC cells were treated with various concentrations of chrysin for 8 h and the cell lysates were subjected to SDS-PAGE to examine the change of indicated protein (*left panels*). Glucose consumption (*middle panels*) and lactate production (*right panels*) in cell culture medium were analyzed. The graph showed the data of at least three independent experiments expressed as means ± SD, the asterisks (*, *p* < 0.05, **, *p* < 0.01, ***, *p* < 0.001, Student’s *t* test) indicated significant inhibition of glucose consumption and lactate secretion after chrysin treatment

Since HK-2 was found to be of high expression in the majority of tested HCC cells, we examined the activity of chrysin in HCC cells with high HK-2 expression. As shown in Fig. 2b, after chrysin treatment, cell proliferation in LM-3, SMMC-7721 and Bel-7402 was substantially inhibited, and more than 50% cell growth inhibition was observed after 72 h treatment. Previous studies reported that tumor cells with high HK-2 expression often displayed high glycolytic phenomenon, we also investigated the effect of chrysin on tumor glycolysis. HCC cells exposed to chrysin (30 μM) showed significantly lower glucose consumption than the untreated. Along with the decrease of glucose uptake, the secretion of lactate, which is the product of tumor glycolysis, was also dramatically decreased (Fig. 2c-e). In accordance with the suppression of tumor glycolysis, in all tested HCC cells, the expression of HK-2 was markedly decreased in a dose-dependent manner (Fig. 2c-e). All these data demonstrated that chrysin displayed an inhibitory effect against cell proliferation and glycolysis in HCC cells via reducing HK-2 expression.

### Chrysin induced HCC cell apoptosis via reducing HK-2 in mitochondria

**Fig. 3:**
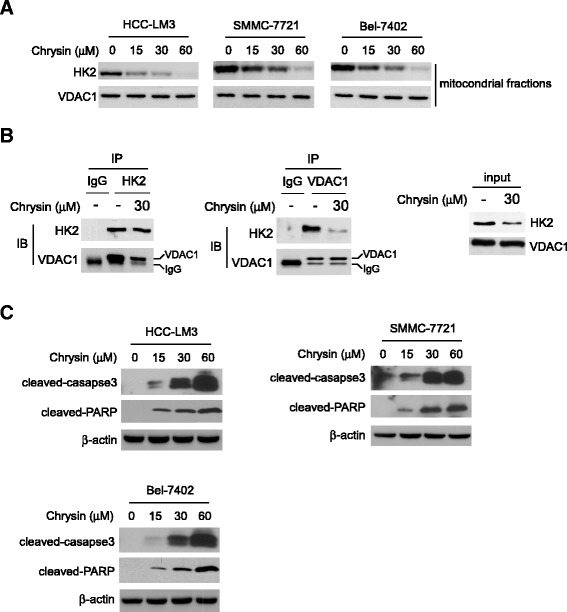
Chrysin induced HCC cell apoptosis by reducing HK-2 in mitochondria. **a**, HCC cells were treated with chrysin for 24 h, the mitochondria fractions was extracted and examined by western blotting to detect the change of indicated protein. **b**, HCC-LM3 cell was treated with chrysin for 24 h and the lysates of mitochondria fractions were immunoprecipitated with HK-2 or VDAC-1 antibodies, then the binding affinity was analyzed with western blotting analysis. **c**, HCC cells were treated with various chrysin for 24 h and cell lysates were probed with indicated antibodies

Generally, HK-2 is located on the out membrane of mitochondria to exert it biological function. In order to further confirm the effect of chrysin on HK-2, we examined the change of HK-2 in mitochondria fractions. As expected, HK-2 in mitochondria was substantially decreased in a dose-dependent manner after exposure to chrysin (Fig. 3a). On the outer membrane of mitochondria, HK-2 interacts with VDAC-1 to form complex to prevent cancer cell apoptosis. Results of immunoprecipitation assay showed that, with the reduction of HK-2 in mitochondria, the HK-2 which bound to VDAC-1 was significantly decreased accordingly (Fig. 3b). Furthermore, as shown in Fig. 3c, cleaved caspase-3 and PARP, which are important markers indicating cell apoptosis, were dramatically elevated, suggesting that the decrease of HK-2 and disruption of HK-2/VDAC-1 interaction caused by chrysin resulted in HCC apoptosis.

### Overexpression of HK-2 attenuated chrysin-mediated glycolysis suppression and cell apoptosis

**Fig. 4:**
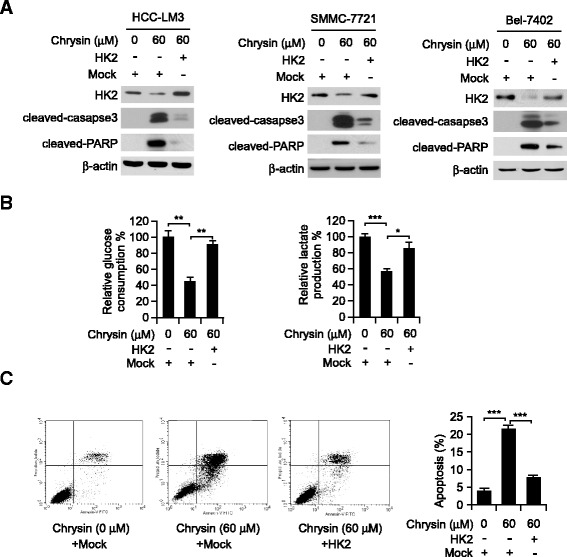
Overexpression of HK-2 impaired the effect of chrysin on apoptosis and tumor glycolysis. **a**, activated caspase-3 and PARP in HCC cell with exogenous HK-2 expression. HCC cells were transfected with pORF-HK-2, 24 h later, the cells were treated with 60 μM chrysin, cell lysates were subjected to SDS-PAGE and probed with indicated protein. **b** and **c**, the effect of chrysin on glycolysis and apoptosis in HK-2 overexpressed HCC-LM3 cells. HCC-LM3 cell was transfected with pORF-HK-2 for 24 h and seeded in 6 well plates for 10 h, then exposed to 60 μM chrysin, tumor glycolysis (**b**) and apoptosis (**c**) was examined at 8 h and 24 h, respectively. **b**, The graph showed the data of at least three independent experiments expressed as means ± SD, the asterisks (*, *p* < 0.05, **, *p* < 0.01, ***, *p* < 0.001, Student’s *t* test) indicated significant difference between different groups. **c**, Representative FACS results of Annexin V-PI double staining were shown (*left panels*), and the graph (right panel) showed the data of at least three independent experiments expressed as means ± SD, the asterisks (***, *p* < 0.001*,* Student’s *t* test) indicated a significant difference

In order to further illustrate the role of HK-2 played in chrysin-mediated activities, HCC cells were transfected with pORF-HK-2 to overexpress HK-2 and then investigated chrysin’s activity. As shown in Fig. 4a, after transfection, reduction of HK-2 caused by chrysin was substantially recovered. With the increase of HK-2, chrysin-mediated suppression of tumor glycolysis was significantly impaired in HK-2-overexpression cells (Fig. 4b). Moreover, in contrast with the mock group, cleaved caspase-3 and PARP were significantly decreased in HK-2 overexpression group, suggesting cell apoptosis induced by chrysin was attenuated (Fig. 4a). Flow cytometry analysis also demonstrated that over 20% HCC cells were undergone apoptosis after treated with 60 μM chrysin, however, the ratio of cell induced apoptosis was significantly decreased in HK-2 overexpression cells. All these results verified chrysin-mediated suppression of glycolysis and induction of apoptosis in HCC cells was closely related to its effect on HK-2.

### Chrysin-induced apoptosis was attributed to activation of Bax

**Fig. 5:**
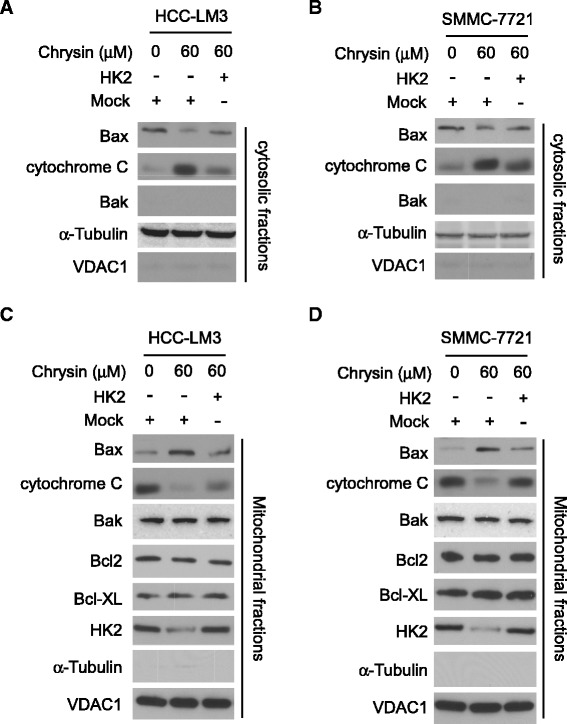
Chrysin induced Bax activation on mitochondrial. HCC cells were transfected with pORF-HK-2, 24 h later, the cells were treated with 60 μM chrysin for another 24 h, and subcellular fractions were prepared and subjected to SDS-PAGE and probed with indicated protein. **a** and **b**, cytosolic Bax, Bak and chrome c expressions in HCC-LM3 cell (**a**) and SMMC-7721 cell (**b**) were tested by western blotting. **c** and **d**, mitochondrial Bax, Bak, Bcl2, Bcl-xl, chrome c, and HK2 in HCC-LM3 cell (**c**) and SMMC-7721 cell (**d**) were tested by western blotting

Involvement of mitochondria in cell apoptosis is considered to be the common mechanism. In order to clarify the mechanism by which chrysin induced HCC cell apoptosis, we investigated the change of pro-apoptotic and anti-apoptotic protein in cytosolic and mitochondria fractions respectively. In HCC-LM3 and SMMC-7721 cells, cytochrome C was significantly increased in the cytosolic fractions after chrysin treatment (Fig. 5a and b). Conversely, Bax, which is a pro-apoptotic protein, was found to be translocated to mitochondria. With the decrease in cytosolic, its expression in mitochondria was dramatically increased. Other proteins involved in apoptosis regulation such as Bak, Bcl-2, Bcl-xL had no obvious change in chrysin treated cells (Fig. 5a-d). In HK-2 overexpression cells, the translocation of Bax from cytosolic to mitochondria was substantially attenuated, and the cytochrome C released from mitochondria into cytoplasm was also impaired, indicating that cell apoptosis induced by chrysin was attributed to the Bax activation on mitochondria.

### Chrysin restrained HCC xenograft growth in vivo

**Fig. 6:**
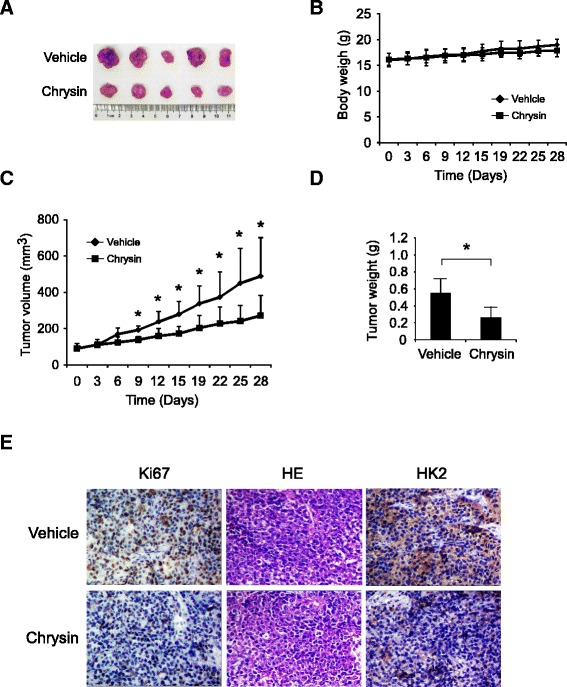
Chrysin inhibited HCC-LM3 xenograft growth in vivo. Nude mice with HCC-LM3 xenograft were randomly divided to groups when tumor volume reached 50 to 100 mm^3^. 30 mg/kg chrysin was administrated three times weekly by intraperitoneal injection. **a**, photograph of tumors in vehicle and chrysin-treated group; **b**, the change of body weight of tumor bearing mice; **c**, tumor growth curve in vehicle and treated group; **d**, tumor weight in vehicle and chrysin group; **e**, tumor tissues were subjected to immunohistochemistry staining with indicated antibodies to detect the change of HK-2, Ki67 after chrysin treatment

To validate the antitumor activity of chrysin against HCC, the efficacy of chrysin was assessed in HCC-LM3 xenograft model. As shown in Fig. 6a-d, comparing with the vehicle group, tumor growth in chrysin group was significantly suppressed. In the end of experiment, the tumor volume of vehicle group had reached about 500 mm^3^, while the average tumor volume of chrysin group was around 200 mm^3^. Meanwhile, no obvious toxicity was observed as evaluating the change of body weight. Immunohistochemistry analysis of chrysin-treated tumor tissue demonstrated the expression of HK-2 in tumor tissue was substantially decreased after chrysin treatment, which confirmed the effect of chrysin on HK-2 in vivo (Fig. 6e). Ki-67 is a well-known marker to represent the potential of cell proliferation, Ki67 in chrysin group was substantially decreased in contrast with the vehicle group. With the suppression of glycolysis by reducing HK-2 in tumor tissue, energy supply to sustain tumor growth was blocked, and the proliferative capability of tumor cell was weakened.

## Discussion

In present study, our results demonstrated that chrysin had profound potency against HCC in vitro and in vivo. Mechanism investigations revealed that HK-2 played a pivotal role for chrysin to exert its activity in HCC. With the reduction of HK-2 after chrysin treatment, the glycolysis in HCC was markedly inhibited. Except the effect on HCC glycolysis, HK-2 was also proved to be involved in chrysin-induced apoptosis. Exposure to chrysin resulted in the decrease of the interaction between HK-2 and VDAC-1, which caused the release of pro-apoptotic proteins from mitochondrial and induced tumor cells to undergo apoptosis.

The expression of HK-2 was reported to be elevated in many cancers and was considered a predictive marker of poor prognosis of HCC [23, 24], breast [25], and gastric cancer [26]. Consistent with previous report, HK-2 expression was detected to be of high rate in most tested HCC cell lines and tumor tissue in contrast with the normal hepatic cell and tissue in our studies (Fig. 1). Dai W et al. demonstrated that HCC cells with high HK-2 expression displayed high aerobic glycolysis, as indicated by increased glucose uptake and lactate production [27]. After chrysin treatment, with the reduction of HK-2, glucose consumption and lactate production in HCC cells were dramatically decreased (Fig. 2). Further investigations revealed that the suppression caused by chrysin was substantially attenuated after overexpression of HK-2, suggesting HK-2 played an important role in chrysin-mediated glycolysis suppression. Metabolic control analysis of HCC cell lines demonstrated that the main control of glycolytic flux was exerted by HKs [28]. Along with the increase of HK-2, altered glucose metabolism often conferred cancer cells resistance to chemotherapy, it was evidenced that the sensitivity of tumor cells to chemotherapy were substantially enhanced via glycolysis inhibition by targeting HK-2 [29, 30]. Given the activity of chrysin against HK-2 and glycolysis, we thought chrysin had the potential to strengthen the efficacy of other chemotherapies.

Previous studies had shown that induction of apoptosis was a key molecular mechanism responsible for the antitumor activity of chrysin. It has been shown that chrysin caused non-small cell lung cancer A549 to undergo apoptosis via the increase of the Bax/Bcl-2 ratio and activation of caspase-3 and −9 [31]. Moreover, chrysin acted as a histone deacetylase (HDAC) inhibitor to induce apoptosis in melanoma A375 by decreasing HDAC targeted gene such as Bcl-xL, Survivin and increasing the level of caspase-3 protein [32]. It also has been depicted in many studies that induction of ROS and depletion of GSH played a significant role in apoptosis induced by chrysin [33, 34]. Different from previous report, no obvious change of anti-apoptotic protein, such as Bcl-xL and Bcl-2 was observed in our study. Our date showed that chrysin-induced apoptosis was mainly attributed to the reduction of HK-2. By positioning itself on the outer membrane of mitochondria, the formation of VDAC-1 and HK-2 complex modulated the integrity and permeability of mitochondria membrane. With the decrease of HK-2 in mitochondria, the interaction between HK-2 and VDAC was disrupted, which resulted in dramatic increase of membrane permeability and release of pro-apoptotic enzyme, such as cytochrome C. Bax in cytoplasm was significantly decreased after chrysin treatment, but Bax located on mitochondria was substantially increased, implying that the majority of Bax was transferred from cytoplasm to mitochondria after HCC cells exposed to chrysin. It was found recombinant Bax induced permeability in liposomes containing VDAC [35], and Bax-activated cytochrome C release was blocked by intracellular microinjection of anti-VDAC antibodies [36]. Moreover, electrophysiological studies revealed that Bax and VDAC combination gave rise to 4 and 10 times increase in conductance in compare with VDAC and Bax channels alone respectively [37]. Pastorino JG et al. reported that HK-2 competed with Bax for interaction with VDAC, and detachment of HK-2 promoted the binding of Bax to VDAC [38]. Therefore, we speculated that owing to the decrease of HK-2 on mitochondria, Bax was more readily to gain access to VDAC, the formation of VDAC-Bax complex was capable of passing cytochrome C and other pro-apoptotic proteins, which induced cell apoptosis. With HK-2 overexpression, Bax binding to VDAC was hindered, and chrysin- induced apoptosis was significantly impaired.

The regulation of HK-2 expression in tumor cells is complicated. Like other enzymes in glycolytic pathway, HK-2 expression is primarily regulated by c-myc, HIF-1α and p53 [39]. Moreover, microRNAs such as miR-143 and miR-155 were evidenced to be capable of modulating HK-2 expression [40, 41]. Fu B et al. reported that chrysin suppressed HIF-1α expression via reducing its stability and inhibiting its synthesis [42]. In our studies, HK-2 was validated to be decreased and reduction of HK-2 played a pivotal role for chrysin to perform its activity in HCC, but further investigations is needed to elaborate the exact mechanism by which chrysin to affect HK-2 expression.

## Conclusion

In conclusion, the results of this study demonstrated chrysin had profound antitumor activity against HCC via inhibiting tumor glycolysis and inducing cell apoptosis. Different from the mechanism reported by previous studies, reduction of HK-2 was an important underlying mechanism for chrysin to exert its effect on cell metabolism and cell apoptosis. This study provided a novel preclinical rational for chrysin, or its analogue, to be applied for HCC management.

## Abbreviations

ATP: Adenosine triphosphate
CDK: Cyclin dependent kinase
GSH: Glutathione
HCC: Hepatocellular carcinoma
HDAC: Histone deacetylase
HK: Hexokinase
ROS: Reactive oxygen species
VDAC: Voltage-dependent anion channels
